# The development and psychometric evaluation of FABIANA-checklist: a scale to assess factors influencing treatment initiation in anorexia nervosa

**DOI:** 10.1186/s40337-021-00490-w

**Published:** 2021-11-03

**Authors:** Laurence Reuter, Denise Kästner, Justine Schmidt, Angelika Weigel, Ulrich Voderholzer, Marion Seidel, Bianca Schwennen, Helge Fehrs, Bernd Löwe, Antje Gumz

**Affiliations:** 1 Department of Psychosomatic Medicine and Psychotherapy, University Medical Center Hamburg-Eppendorf, Martinistr. 52, W37, 20246 Hamburg, Germany; 2 Department of Psychiatry and Psychotherapy, University Hospital, LMU Munich, Munich, Germany; 3 Schön Clinic Roseneck, Prien am Chiemsee, Germany; 4 Department of Psychiatry and Psychotherapy, University Hospital of Freiburg, Freiburg, Germany; 5 Schön Clinic for Psychosomatic Medicine and Psychotherapy, Bad Arolsen, Germany; 6 Medclin Seepark Clinic for Acute Psychosomatic Care, Bad Bodenteich, Germany; 7 Department of Psychosomatic Medicine and Psychotherapy, Asklepios Westklinikum Hamburg, Hamburg, Germany

**Keywords:** Anorexia nervosa, Duration of untreated illness, Early intervention, Facilitators and barriers, Psychotherapy, Psychometrics, Construct validity

## Abstract

**Background:**

A long duration of untreated illness (DUI) is an unfavorable prognostic factor in anorexia nervosa (AN) and is associated with chronic illness progression. Although previous preventive measures aimed at reducing DUI and thus improving short- and long-term treatment outcomes have been partially successful, a better understanding of the factors involved in the sensitive phase prior to treatment initiation is needed. To date, there is no validated instrument available to assess these factors specifically for patients with AN. The FABIANA-project (Facilitators and barriers in anorexia nervosa treatment initiation) aims at identifying predictors of the DUI in order to target preventive measures better in the future. As part of this project, the FABIANA-checklist was developed, based on a multi-informant perspective and a multimodal bottom-up approach. The present study focusses on the process of item generation, item selection and psychometric validation of the checklist.

**Methods:**

Based upon a previous qualitative study, an initial set of 73 items was generated for the most frequently mentioned facilitators and barriers of treatment initiation in AN. After a process of consensual rating and cognitive pre-testing, the resulting 25-item version of the FABIANA-checklist was provided to a sample of female patients (N = 75), aged ≥ 14 years with AN that underwent their first psychotherapeutic treatment in the last 12 months. After item analysis, dimensionality of the final version of the FABIANA-checklist was tested by Principal Component Analysis (PCA). We evaluated construct validity assuming correlations with related constructs, such as perceived social support (F-SozU), support in the health care system (PACIC-5A), illness perception and coping (BIPQ).

**Results:**

We included 54 adult and 21 adolescent patients with AN, aged on average 21.4 years. Average BMI was 15.5 kg/m^2^, age of onset was 19.2 years and average DUI was 2.25 years. After item analysis, 7 items were excluded. The PCA of the 18-item-FABIANA-checklist yielded six components explaining 62.64% of the total variance. Overall internal consistency was acceptable (Cronbach’s α = .76) and construct validity was satisfactory for 14 out of 18 items. Two consistent components emerged: “primary care perceived as supportive and competent” (23.33%) and “emotional and practical support from relatives” (9.98%). With regard to the other components, the heterogeneity of the items led to unsatisfactory internal consistency, single item loading and in part ambiguous interpretability.

**Conclusions:**

The FABIANA-checklist is a valid instrument to assess factors involved in the process of treatment initiation of patients with AN. Psychometrics and dimensionality testing suggests that experienced emotional and practical support from the primary health care system and close relatives are main components. The results indicate that a differentiated assessment at item level is appropriate. In order to quantify the relative importance of the factors and to derive recommendations on early-intervention approaches, the predictive effect of the FABIANA-items on the DUI will be determined in a subsequent study which will further include the perspective of relatives and primary caregivers.

*Trial registration* Clinical Trials.gov Identifier: NCT03713541: https://clinicaltrials.gov/ct2/show/NCT03713541.

**Supplementary Information:**

The online version contains supplementary material available at 10.1186/s40337-021-00490-w.

## Plain English summary

Early treatment contributes to a more favorable illness course and an improved prognosis in patients with anorexia nervosa (AN). The current study presents the development of the FABIANA checklist, which aims to assess factors which influence duration of untreated illness. The FABIANA checklist was developed on the basis of interviews with patients, their relatives and primary care practitioners. It provides data from the first use of the checklist in a German sample of 75 patients with AN. The results of our study suggest that the FABIANA-checklist is a valid instrument to assess factors involved in the process of treatment initiation. Emotional and practical support from the primary health care system and close relatives were the most consistent components. A follow-up study will investigate the relationship between the FABIANA-items and the DUI in order to guide the conception of effective secondary prevention measures.

**Supplementary Information:**

The online version contains supplementary material available at 10.1186/s40337-021-00490-w.

## Background

Anorexia nervosa (AN) is a serious mental disorder often characterized by variable outcomes, high mortality rates and a high risk for a chronic course of the illness [1, 2]. Only a minority of patients are inwardly motivated to immediately seek specialized treatment [3–5], and it often takes several years for patients to receive appropriate therapy [6–8]. A recent review [6] including 14 studies found a mean duration of untreated illness (DUI) of 29.9 months (range = 6.4 to 39.9 months) in patients with AN. In children under 12 years of age, the DUI averaged 10 months, compared with 35 months in adolescents and adults.

Patients with AN have a better prognosis if evidence-based treatment [12] is provided timely and at early stages of illness [13, 14]. A delayed access to treatment has been associated with higher levels of psychological distress as well as with social and occupational impairment [15, 16]. Ambwani et al. [17] reported e.g. a positive association between disease duration and symptom severity at admission. Patients with a longer duration of illness (defined as > 3 years) had poorer long-term treatment outcomes and showed less improvement in their social or occupational adjustment one year after the end of treatment, compared with a group of patients with a recent onset (< 3 years). The importance of a rapid intervention in patients with AN is further supported by a growing number of studies showing that the maladaptive behaviors in eating disorders become increasingly automated over time and, thus, more resistant to change [18–20].

Most studies divide the DUI into two phases [8–10]. The first phase describes the period between the initial manifestation of the disease and the patient's first contact with primary health care. This phase, which constitutes the larger part of DUI [4, 11], is characterized by a high degree of inner ambivalence of the patient with regard to the disease and the therapy. In a favorable case, the patient develops an acceptance of the illness and a motivation for treatment. The decision to seek help marks the transition to the second phase, which is mainly characterized by waiting for a referral to specialized treatment. Parents or relatives play an important role in the initial phase of the disease. Since they often live closely with young patients, they might address the AN at an early stage of the illness and accompany the patient to primary health care. The waiting time until referral to specialized care, on the other hand, depends more on the structural conditions and interdisciplinary cooperation within the respective health system.

Preventive interventions aiming at facilitating help-seeking behavior and optimizing pathways to specialized care lead to divergent outcomes [21–23]. In a previous study, members of our research group implemented a systemic public health intervention (psychenet), which aimed at facilitating the early recognition and treatment initiation in patients with AN [9, 24]. The implementation of a wide-range of preventive measures, such as disseminating information about AN, developing specialized treatment programs, establishing a multidisciplinary health network and setting up a specialized outpatient clinic, however, did not lead to a significant reduction of the DUI [21]. In the UK, the FREED-up program [10, 22] addresses the waiting time between the first contact with primary care and the start of a specialized treatment. Through person-centered contacts, referred patients receive information about their eating disorder and are given the opportunity to talk about fears and concerns regarding treatment. The aim is to build up motivation for a subsequent assessment and specialized AN-treatment [10, 22]. In the recently published FREED-up study [22], it has been shown that FREED participants had a significantly shorter DUI, faced shorter waiting times and had higher rates of treatment uptake in comparison to TAU. When FREED was optimally implemented (e.g. immediate specialist evidence-based assessment and treatment directly at help-seeking), which was the case for 56.5% of the total sample (N = 278), the reduction of DUI was pronounced, underlining the importance of well-interlinked care. However, the time span from the onset of the disease to the first contact with a specialist was not targeted by FREED. Moreover, a significant proportion of potential patients were not fully reached by the program, i.e. they dropped out of the program between referral from primary care and assessment. The authors attribute this to the ongoing ambivalence of many patients with AN regarding treatment initiation [22]. The results of this promising program suggest that the focus of interventions on service-related factors, might not be sufficient to address DUI in this highly ambivalent group of patients.

In order to target preventive measures better at the different phases in the process of treatment initiation, it is crucial to assess which factors are involved in the process of treatment initiation. Among the barriers involved in treatment initiation in patients with eating disorders (ED), Innes et al. [25] included patient-related factors such as stigma, unhelpful past treatment experiences, fear of change or low motivation. Service-related barriers are predominantly service availability, service restrictions and treatment costs [25]. The authors of this literature review, which included 11 studies, point out that the heterogeneity of the samples included (exclusively ED diagnosis or subclinical sample; female vs. mixed gender; adults vs. adults and adolescents; different ethnic background) and the methods applied (qualitative vs. quantitative methods, retrospective vs. prospective; dichotomous vs. dimensional assessment) do not allow comparisons between the studies or a quantification of the barriers assessed. The diagnostic representation of the different types of ED  including binge-eating disorder (BED), bulimia nervosa (BN) and AN was furthermore unbalanced, with an underrepresentation of patients with AN in the studies included.

According to the assessment of the barriers, five studies used a qualitative methodology with an open question about inhibiting or helpful factors in the process of treatment initiation. When checklists or rating scales were used, items were mainly derived from previous research on help-seeking behavior in patients with different mental health issues. The authors concluded that the “few instruments that have quantitatively assessed barriers have developed instruments without a clear justification for the items and importantly the instruments lacked psychometric rigor” (p. 18). None of the studies reported data on reliability or validity of the measures employed nor provided psychometrical support for the dimensionality of the applied factors or subscales [25].

Subsequent to the literature review, Innes et al. [26] provided psychometric data and analyzed the construct validity of the “Perceived Barriers to Psychological Treatment Scale” [27] for a sample (N = 708) of female patients aged > 14 years with disordered eating behavior. The sample included 53.81% of patients within the clinical range of ED, and about 20% of the clinical subgroup fulfilled the criteria for an AN. Factor analysis yielded a seven-factor solution including—sorted in descending order of importance—access to services, time constraints, stigma, lack of motivation, negative evaluation of treatment, emotional concerns, participation restrictions and access restriction. The overall score of perceived barriers was negatively correlated to general help-seeking intentions (r = − .28) and to intentions to seek help from a mental health professional (− .19). Although this questionnaire has good psychometric values, it registers general barriers to seeking psychological treatment. It does not register disorder-specific barriers (e.g. fear of gaining weight). This might be reflected by the low correlations with help-seeking behavior in the population of patients with disordered eating behavior.

In a more recent paper, Ali et al. [28, 29] assessed and quantified barriers to help-seeking among a clinical sample of patients with different types of ED. The 15 barriers, which had been extracted from a literature review [28] were systemized in the “Barriers toward seeking help for eating disorders questionnaire” (BATSH-ED). The BATSH-ED covers a broad range of barriers for help-seeking behavior and mainly refers to patient-related barriers, such as internal attitudes, experiences and beliefs regarding the ED. These include aspects such as denying the disease or not perceiving its severity, not wishing to be a burden for others, feeling ashamed about the ED or the fear of losing control. While these and other aspects, such as a lack of knowledge or information about eating disorders, are suitable for potential preventive measures, other aspects, such as “previous negative experiences” or “comorbidities” cannot be addressed by preventive measures. The few barriers related to the health system or the social environment are described by more general items, so that it seems rather difficult to derive concrete measures here as well.

In addition to the question whether the barriers of the BATSH-ED are suitable for deducing preventive measures, it also remains questionable as to what extent the measure, which was developed for ED in general, is suitable to specifically address AN-specific factors. The use of the BATSH-ED in a population with different eating disorders (N = 291) showed, despite the small size of the AN-subsample (N = 10), differences with regard to the importance of the barriers in treatment initiation in the ED-subgroups [29]. Patients with AN were more frequently embarrassed about their problems, were more afraid of treatment or saw treatment as a sign of weakness. A further limitation of the BATSH-ED is, that it includes only those barriers that were mentioned in the previous literature review. Aspects that might be relevant to patients but have not yet been researched are not addressed by the checklist. Furthermore, it does not take into account the factors that facilitate help seeking behavior, nor includes the perspective of primary caregivers and relatives.

In view of the limitations of the existing measures, the aim of the FABIANA-study (Facilitators and barriers in anorexia nervosa treatment initiation, [30]) was to develop, in a first step, a checklist to assess factors (facilitators and barriers) of treatment initiation in patients with AN using a multi-method and multi-informant approach.

To achieve a wide spectrum of factors involved in treatment initiation in patients with AN we used semi-structured interviews (N = 22) with patients, relatives and primary care physicians [31]. Using Grounded Theory we identified facilitators and barriers within the patient, the social environment, the health care system and society. Based upon the most prominent factors (see Additional file 1) of this study [31] the FABIANA-checklist was developed.

The present study reports on the process of item construction and item selection of the FABIANA-checklist and examines the psychometric properties and dimensionality of the checklist among a sample of German patients with AN. It further quantifies factors involved in AN treatment initiation as experienced by patients.

The long-term goal of the FABIANA study is to quantify the magnitude of the effects of the factors assessed with the FABIANA-checklist on the DUI (upcoming study) with the intention of giving recommendations to guide the conception of effective secondary prevention approaches.

## Method

**(1) The development of the FABIANA-Checklist**

Instrument and item generation were based on a recommended procedure for mixed-method studies [32]. Details and data on item generation, item evaluation and item selection can be found in the Additional file 1.

### Item generation

For item generation, we considered facilitators and barriers which had been mentioned in at 25% of the interviews (N = 22) of our previous qualitative study [31] and/or had more than 10 codings and had been considered as addressable through secondary prevention measures. The latter rating was provided after consensual discussion in the research team, consisting of one professor of psychosomatic medicine and psychotherapy, two post-doctoral clinical psychologists and one clinical psychologist. Two frequently named factors were excluded due to difficult modifiability, namely “(not) living, being or eating alone”; “good personal connections” to somebody working within medical services. Two further factors ("waiting time and availability of treatment"; "fit between individual patient and service setting") were considered to be too unspecific and exclusively related to primary prevention and, therefore, excluded.

Based upon prototypical quotes from our previous qualitative study [31], items were formulated for each of the selected 21 factors. It was ensured that the items represented all facets of each factor. As the content of some factors was more heterogeneous than others, the number of items per factor ranged from 1 to 7.

### Item selection

The first set of 73 items was rated independently by the research team named above. Raters considered clinical relevance, addressability through secondary prevention measures and comprehensibility of each item and assigned a composite score from 1 to 3 points, with 1 = inappropriate item recommended for exclusion, 2 = potentially appropriate item requiring modifications to improve understandability, and 3 = very appropriate item recommended for inclusion in the checklist.

Interrater-reliability (Fleiss’ Kappa for ordinal data) was moderate (*k* = 0.45; 95% CI [0.39, 0.53]). The agreement was substantial for item inclusion (*k* = 0.60; 95% CI: [0.51, 0.70]), moderate for item exclusion (*k* = 0.47; 96% CI [0.35, 0.54] and fair for potentially suitable items (*k* = 0.32; 95% CI [0.23, 0.43]).

Items with a full agreement for inclusion (mean score of 3 points; *n* = 15) were included in the checklist. Items with both a mean score < 2 points and single ratings ≤ 2 points (*n* = 20) were excluded from the checklist. For the remaining items (*n* = 38), group discussions were held in the research group until consensus regarding inclusion (n = 15) or exclusion (n = 13) was reached. Suggestions about alternative formulations were considered and it was ensured that each factor was represented by at least one item. If necessary, the wording of some items was revised. A total of 30 items was considered for the first version of the checklist, which subsequently underwent cognitive pre-testing.

### Cognitive pre-tests

The 30-item FABIANA-checklist underwent cognitive pretesting in a sample of 9 adult female patients with AN (average age 22.8 years, *SD* = 5.6, [18, 35]) recruited in a specialized inpatient unit for AN treatment (inpatients *n* = 8, day-care *n* = 1). The sample had an average BMI of 16.8 kg/m^2^ (*SD* = 1.9) and was predominantly diagnosed with a restrictive type of AN (N = 8). Exclusion criteria were a participation in our previous qualitative study or insufficient German language skills. After informed consent, patients completed the FABIANA checklist, which took on average about five minutes (*M* = 04:25).

Patients were asked to rate the extent each aspect applied to their personal experiences in the period from illness onset to the start of a specialized psychotherapeutic treatment on a 5-point Likert scale  (1 = strongly disagree to 5 = strongly agree). After completing the checklist, personal interviews addressed the relevance (“How important was this aspect for initiating the psychotherapeutic treatment?”), comprehensibility (“How comprehensible is the item?”) and the recallability (“How well do you remember the described aspect?”) for each item and answers were rated on a 3-point scale, with 1 = not at all, 2 = partially and 3 = very much.

The interrater reliability (ICC with mixed effects model and absolute agreement) was good (ICC = 0.89; 95% CI [0.85, 0.92]). With regard to the assessed relevance, the 30 items were normally distributed around the mean value of 1.96 (*SD* = 0.47). Average comprehensibility (*M* = 2.88, SD = 0.17) and recallability (*M* = 2.95, *SD* = 0.08) were very high.

After discussion of the results in the research group, 5 items were excluded from the checklist. Decisive was a combination of low relevance (< 2 points), a similarity to other items of the same factor or an unspecific formulation and thus the difficulty of deriving preventive measures. The resulting FABIANA-checklist with 25 items underwent psychometric validation.

**(2) Psychometric analysis and validation of the FABIANA-checklist**

The FABIANA-study has been registered (NCT03713541) and ethical approval was obtained prior to recruitment (PV5108).

### Data collection

Data for the psychometric validation of the FABIANA-checklist was collected between July 2018 and June 2019 in 11 cooperating in- and outpatient centers specialized in the psychotherapeutic treatment for ED. Inclusion criteria were an age ≥ 14 years, female gender and typical or atypical AN diagnosis. We included patients who were either currently in their first AN treatment or who had initiated their first psychotherapeutic AN treatment within the last 12 months. Psychotherapeutic treatments were defined by a minimum duration of seven days in an inpatient setting or five consecutive sessions in an outpatient setting. We planned to include at least four participants for each item of the FABIANA-checklist [30, 33].

After obtaining written informed consent from the patients and their legal guardians, eligible patients completed the assessment battery, which consisted of sociodemographic data, the 25-item version of the FABIANA-checklist and the questionnaires used for construct validation. Clinical data such as the current treatment diagnosis, Body Mass Index (BMI, kg/m^2^) and the date of treatment initiation were provided by the treating specialist. Patients subsequently participated in structured clinical interviews (SCID-5-CV, [34]) to validate the AN diagnosis and to assess AN subtypes and comorbidity.

Particular attention was paid to the assessment of illness and weight history. To ensure a higher recall accuracy anchor examples, as for example relevant events in the patients’ life, were marked on a timeline at the respective age of the patient. The interviewer repeatedly referred to this timeline in order to support patients in recalling their symptoms at a given time point. Age of onset (AOO) was defined as the age (in years) of the patient at the moment when the criteria for AN were fully met for the first time. The DUI was defined as the difference between the date of illness onset and the date of first treatment initiation (in years).

For sample characteristics we considered descriptive data. To account for differences between adults and adolescents regarding DUI and AOO we used simple t-tests. All calculations were performed with SPSS 27.

### Item analysis and dimensionality of the FABIANA-checklist

For item analysis, we considered descriptive data (*N, mean, standard deviation, range*), graphical distributions of the raw values (histogram, Q-Q plot), skewness, kurtosis and item difficulty. For item discrimination we used Pearson’s correlation coefficient (r^it^), i.e. the correlation between the single item and the global score. Internal consistency was evaluated by Cronbach’s alpha. Before performing scale statistics, polarity of negatively poled items was reversed (see also annotations in Table 2 and 3). We used consensual group discussions to decide upon inclusion or exclusion of the items in the final version of the checklist. The aim was to obtain an economic and internally consistent scale (Cronbach's α > 0.70) that captures a broad spectrum of factors involved in treatment initiation of patients with AN.

Dimensionality of the FABIANA-checklist was tested by Principal Component Analysis (PCA). Data suitability test included the Kaiser–Meyer–Olkin (KMO) criterion and Bartlett’ test for sphericity. We considered components with eigenvalues ≥ 1 [35] and tested for varimax, quartimax and equamax rotation on data. We considered and reported factor loadings of > 0.30. In the case of cross-loadings on multiple components, we considered the loading that showed the best interpretability. For each component the explained variance, the eigenvalues and the internal validity (Cronbach's alpha) are given. At item level correlations between the item and the respective component (r^it^) and changes in the internal validity of the component when the item is deleted are provided.

### Construct validity of the FABIANA-checklist

We evaluated construct validity of FABIANA-checklist by testing hypotheses regarding correlations with related well-established constructs, namely to collaborative support from health care providers, perceived social support and illness-perception. All assumptions for construct validation were made for each item separately. Expected correlations are shown in Table 3.

We expected positive correlations between the FABIANA-items related to experienced support from health care members (e.g. general practitioners, gynecologist, psychiatrist) and the global score of the *Patient Assessment of Chronic Illness Care questionnaire*. The PACIC-5A [36] is a brief self-administrated instrument to assess whether the patients were provided with patient-centered collaborative care prior to their psychotherapy. The PACIC-5A relates to the chronic care model [39] and measures the extent to which professionals tried to induce behavioral changes in patients. The 5A approach is evidence-based, has achieved widespread acceptance and is considered the most appropriate and psychometrically robust instrument assessing patient experience with chronic disease care [40]. The global score includes the assessment of present behavior, patient counselling, collaborative agreement with the patient about realistic goals, assisting the patient during her lifestyle changes, and frequent follow-ups.

We further assumed positive correlations between the FABIANA-items, which include concern or concrete support from relatives or the social environment with the total score of the *Social Support Questionnaire* (F-SozU [37]). The unidimensional short version of the F-SozU assesses general perceived social support. For comparability, patients were asked in the introduction to refer to the period between the onset of AN and the initiation of specialized psychotherapeutic treatment. The 14 items of the F-SozU are rated on a scale from 0 = did not apply to 4 = did fully apply. The total score results from the mean value of all items. The F-SozU shows good psychometric properties and a good internal consistency (Cronbach's α = 0.94).

For the FABIANA-items relating to societal factors, as for example the influence of media or stigmatization, we assumed correlations with subscales of Brief illness perception Questionnaire. The *Brief Illness Perception Questionnaire* (BIPQ [38]) assesses patients’ illness representations, including the degree of understanding of the illness, the perceived personal and treatment control, the experience of symptoms, concerns about the illness and emotional effects of the illness on a numeric rating scale (0–10). For construct validation, we considered positive correlations between the FABIANA-items (see Table 3) and personal control, treatment control and illness comprehensibility. In addition, we included the open-ended BIPQ item on subjective illness causes, in which patients are asked to record the three major causes of their AN. Since we were particularly interested in the influence of media, we assessed whether or not (dichotomous variable) answers regarding the influence of media were given in the open-ended item of the BIPQ.

Construct validity was tested with bivariate correlations. We report correlation coefficients and *p-*values for one-tailed testing (α < 0.10). Correlation coefficients were interpreted based on Cohen’s d with *d* =  < 0.30 as a small, *d* = 0.30–50 medium and *d* > 0.50 as large [41].

## Results

### Sample characteristics

We recruited 75 female patients with AN of which 54 were adult and 21 were adolescent. Mean age was 21.4 years (*SD* = 7.35 [14.0, 61.0]). Most patients (89%) were diagnosed with typical AN and 77% presented the restrictive AN-subtype. The mean BMI was 15.5 kg/m^2^ *(SD* = 1.96 [10.6, 23.0]). Most patients (77%) had at least one comorbid mental disorder, diagnosed by SCID-5-CV interview [34]. Table 1 shows the sample characteristics.

**Table 1:** Sample characteristics

	*n* = 75	Adults (*n* = 54)	Adolescents (*n* = 21)	*F, p*
Age; *M* in years, (*SD,* [*range*])	21.4 (7.35, [14, 61])	23.69 (4.47, [18, 61])	15.52 (1.17 [14, 17])	
*Setting; n (%)*				
Inpatient	70 (93.3)	51 (94.4)	19 (90.5)	
Outpatient	5 (6.7)	3 (5.6)	2 (9.5)	
*Diagnosis – DSM V; n (%)*				
Typical anorexia nervosa (F50.0)	67 (89.3)	47 (87.0)	20 (95.2)	
Atypical anorexia nervosa (F50.1)	8 (10.7)	7 (13.0)	1 (4.8)	
*AN subtype; n (%)*				
restrictive	58 (77.3)	40 (74.1)	18 (85.7)	
binge-purging	17 (22.7)	14 (25.9)	3 (14.3)	
DUI; *M* in years, (*SD,* [*range*])	2.25 (4.33, [.15, 19.6])	2.93 (4.94, [.15, 19.6])	.49 (.59, [− .17, 2.29])	5.083*
AOO; *M* in years, (*SD,* [*range*])	19.15 (5.18, [12, 41])	20.75 (5.26, [12, 41])	15.03 (1.07, [13, 17])	24.182***
BMI; *M* in kg/m^2^, (*SD,* [*range*])	15.5 (1.96, [10.6, 23.0] /)	15.5 (1.91, [10.6–21.1])	15.0 (2.12, [13.0, 23.3])	
*Comorbid mental disorder; n (%)*				
None	17 (22.6)	10 (18.5)	7 (33.3)	
One	42 (56.0)	23 (63.0)	8 (38.1)	
Two or more	16 (21.4)	10 (18.5)	6 (28.6)	
*Comorbid personality disorder; n (%)*				
None	70 (93.3)	49 (90.7)	21 (100)	
One	5 (6.7)	5 (9.3)	0 (0)	

DUI = Duration of untreated illness; AOO = Age of onset; F-values for simple t-tests comparing adults to adolescents, with * for *p* < .05 and *** for *p* < .001 (two-sided)

In our sample we observed an average DUI of 2.25 years (*SD* = 4.33, [− 0.17, 19.59]. Note regarding negative DUI: one patient with atypical AN fully met the diagnostic criteria for AN after treatment initiation). DUI in adolescent patients (*M* = 0.49 years, SD = 0.59 [− 0.17, 2.29]) was significantly shorter compared to adults (*M* = 2.93 years, *SD* = 4.94, [0.15, 19.59], *p* = 0.027). A treatment initiation within the first year after illness onset could be observed in 64% of patients, predominantly in the first three quartiles (Q1: 17.3%, Q2: 25.3%, Q3: 17.3%, and Q4: 4%). A DUI between one and three years was found in 17.4% of the patients, 13.3% had a DUI between 3 and 7 years and 5.3% had a DUI between 18 and 20 years. The average AOO was 19.15 years (*SD* = 5.18, [12, 41]). Adolescents had significantly lower AOO compared to adults (with *M* = 15.03; *SD* = 1.07 [13.0, 17.0] and *M* = 20.75, *SD* = 5.26, [12.0, 41.0]; *p* = 0.000).

### Item analysis and dimensionality

Results from item analysis are shown in Table 2. For each item, the total range between the minimum and maximum value (1–5) was used. After consensual group discussion, 7 items were excluded. Reasons for exclusion were poorer item-total correlation values compared to another item of the same factor (item a: *r*^it^ = 0.17 vs. item 2: *r*^it^ = 0.42, item b: *r*^it^ = − 0.08 vs. item 3: *r*^it^ = 0.03 and item d: *r*^it^ = 0.13 vs. item 8: *r*^it^ = 0.50). Item c, e, f and g showed r^it^ values around or below 0. As these factors were also judged to be less modifiable we opted for the exclusion of these items and the respective factors (e.g. the factor “somatic symptoms and/or exacerbation and personal breaking point reached”). Excluding these 7 items increased internal consistency of the checklist (Cronbach's alpha) from 0.70 to 0.77. The final version of the FABIANA-checklist included 17 of the factors extracted from the qualitative interviews [31], represented by 18 items.

**Table 2:** Item characteristics of the FABIANA-checklist

FABIANA-checklist (N = 18)		*N*	*M*	*M*^*1*^	*SD*	Adults		Adolescents		*p*	Item analysis			25 items		18 items	
						*M*	*SD*	*M*	*SD*		*SK*	*KT*	*DIF*	*r*^*tt*^	*α*	*r*^*tt*^	*α*
1	People from my social environment have never addressed my anorexia, or have done so too late.^1^	75	3.1	2.9	1.17	3.0	1.18	3.4	1.12		.24	− .63	.47	.06	.71	.15	.77
2	There was at least one person in my social environment who understood at an early stage of the illness that I needed professional help (e.g. from a doctor, a psychotherapist or an counselling center)	75	3.6		1.51	3.7	1.51	3.3	1.53		− .63^3^	− 1.16	.65	.42	.67	.34	.75
3	It helped me to look at or read articles regarding the successful treatment of anorexia or the recovery of other people with anorexia (e.g. books, reports, social media)	73	3.0		1.34	3.1	1.39	2.8	1.20		− .08	− 1.12	.50	.03	.71	.09	.77
4	My social environment often expressed concern about my anorexia	75	4.2		1.02	4.1	1.01	4.3	1.06		− 1.00^3^	.18	.79	.50	.68	.47	.75
5	At least one person from my social environment supported me practically in treatment initiation (e.g. arranged or accompanied me to medical appointments)	75	4.1		1.41	4.1	1.41	4.1	1.46		− 1.31^3^	.18	.78	.24	.69	.18	.77
6	My environment has encouraged me to take up treatment	74	4.5		.98	4.5	.93	4.5	1.15		− 2.20^3^	4.78	.86	.50	.68	.44	.75
7	My relatives or persons I relate to closely informed themselves on the subject of anorexia (e.g. read books, researched on the internet, visited a counseling center or a doctor ^2^)	75	2.7		1.59	3.3	1.40	4.1	1.11	*	− .35	− 1.18	.63	.31	.69	.26	.76
8	I had a doctor^2^ who arranged that I received appropriate treatment	75	3.2		1.46	3.2	1.44	3.2	1.55		− .09	− 1.38	.55	.50	.67	.53	.74
9	I had a doctor^2^ who recognized my anorexia at an early stage of the illness	75	2.3		1.40	2.2	1.42	2.4	1.36		.73^4^	− .77	.31	.47	.67	.55	.74
10	I had a doctor^2^ who told me unambiguously that I had anorexia	75	3.1		1.63	3.1	1.61	3.0	1.72		− .13	− 1.60	.51	.34	.68	.45	.75
11	I had a doctor^2^ who dealt badly with my difficulties concerning food, body-shape or weight (e.g. did not take my complaints seriously or trivialized them).^1^	75	2.7	3.3	1.59	2.7	1.56	2.5	1.69		− .38	− 1.44	.58	.33	.68	.28	.76
12	After my anorexia was recognized. I had regular appointments with a doctor^2^	74	3.3		1.43	3.2	1.40	3.8	1.45		− .33	− 1.22	.58	.39	.68	.40	.75
13	I had a doctor^2^ I trusted	75	3.4		1.48	3.3	1.43	3.4	1.63		− .39	− 1.25	.59	.47	.67	.51	.74
14	I had a doctor^2^ with high competence in the field of eating disorders	75	2.7		1.38	2.6	1.37	2.9	1.41		.18	− 1.24	.41	.49	.67	.55	.74
15	I had a doctor who collaborated well with my other practitioners^2^	74	2.5		1.45	2.5	1.42	2.4	1.57		.60^4^	− .96	.36	.49	.67	.52	.74
16	It was difficult for me and/or my relatives or persons I relate to closely to find out whom I could consult best to get help.^1^	75	3.3	2.7	1.40	3.3	1.41	3.2	1.38		.33	− 1.16	.42	.20	.70	.22	.76
17	I believed that undergoing psychotherapy was a sign of weakness.^1^	74	3.3	2.7	1.43	3.3	1.44	3.5	1.44		.28	− 1.25	.42	.11	.70	.14	.77
18	By comparing with girls or women in the media (e.g. television. internet. social media) I considered a certain diet (e.g. a very restricted diet), a very slim figure or a very low weight to be normal so that I didn’t feel the need for treatment.^1^	75	3.8	2.2	1.32	3.8	1.31	3.7	1.35		.68^4^	− .79	.30	.17	.70	.12	.77
*Excluded items (N* = *7)*																	
a	My social environment did not understand my illness (e.g. "Just start eating again").^1^	74	3.7	2.3	1.03	3.7	.98	3.7	1.17		.45	− .58	.32	.17	.70		
b	It helped me to exchange ideas with people who had already undergone psychotherapy	73	2.8		1.61	2.8	1.65	2.8	1.54		.12	− 1.62	.45	− .08	.72		
c	At least one person from my social environment has frequently blamed me for my anorexia nervosa.^1^	75	2.7	3.3	1.43	2.7	1.50	2.8	1.26		− .26	− 1.24	.57	.02	.71		
d	After my anorexia was diagnosed, I was in medical treatment for a long time without getting a clear recommendation for psychotherapy.^1^	73	1.8	4.2	1.09	1.7	.97	2.1	1.35		− 1.55^3^	1.79	.81	.13	.70		
e	I was only ready for treatment as my (physical or mental) breaking point was reached	74	4.0		1.18	4.1	1.18	3.8	1.20		− 1.07^3^	.30	.75	− .15	.72		
f	I was afraid that the treatment would not be compatible with other things important to me (e.g. school, job, children).^1^	75	4.4	1.6	1.06	4.4	1.14	4.3	.86		1.61^4^	1.72	.16	.08	.70		
g	Without my physical symptoms and complaints, I would not have undergone psychotherapy	73	4.2		1.11	4.1	1.22	4.4	.61		− 1.44^3^	1.37	.80	− .01	.71		
**Cronbach’s alpha (scale)**														**.70**		**.77**	

^1^Items were inverted for item analysis

^2^Patients are asked in the introduction of the questionnaire, to refer to the physicians they consulted before being referred to specialized care

^3^Left-skewed item

^4^Right-skewed items

*N* = number of participants who completed the item; *M* = mean value, *M*^1^ = Mean value after item inversion, *SD* = standard deviation*, p*-values for simple t-tests comparing adults to adolescents, *p < .05, *SK* = skewness, *KT* = kurtosis, *DIF* = item difficulty (0–1), r^it^ = item-total correlation, *α* = Cronbach’s alpha when item is deleted,

PCA was performed after item selection. Sample size (*N* = 75) was adequate (KMO = 0.72) and Bartlett's test of sphericity indicated that data structure was appropriate for running PCA (χ^2^ = 307.26; *p* < 0.001). A scree-plot yielded empirical justification for retaining six factors with eigenvalues > 1, which accounted for 62.46% of the total variance. Among the tested rotations, the varimax-rotated solution was the most interpretable. Table 3 indicates results of the PCA. At the component level, the internal consistency for the first two factors was in an acceptable range (Cronbach’s α = 0.67–0.79). The first component explained 23.33% of the variance. The eight items included aspects of the health care system that patients experienced as supportive and helpful (e.g., trust in the treating physician, the treating physician's competence in the area of eating disorders or good cooperation between different physicians). The second component, explained 9.98% of the variance and included three items related to emotional and practical support from relatives. On the third component (“Need and searching for orientation and help”), inconsistencies in terms of factor loadings manifested in negative internal consistency. On the one hand, this component reflected the disorientation of the patients and their relatives, not knowing whom to consult for help, and on the other hand it included the patient’s attempt to gather information about helpful treatment processes from books or social media. The fourth component (7.71%) included items showing that AN was taken seriously (not trivialized) by the patients or their social environment (e.g. AN was not addressed too late or at least one person of the environment understood the need for professional help). Internal consistency of this factor was however insufficient (Cronbach’s α = 0.42). Component four and five showed single-item loadings, relating on relatives who informed themselves about AN (6.76%) and the absence of negative media influence (5.82%).

**Table 3:** Principal component analysis and construct validation of the FABIANA-checklist (18 items)

Components (Eigenvalue)	Items (Nr.)	Principal Components Analysis								Construct validity	
		1	2	3	4	5	6	*r*^*it*^	*α*	Assumptions	*r, p*
1 Primary care perceived as supportive and competent (4.20)	I had a doctor^2^ I trusted. (13)	.84						.61	.75	PACIC-5A	.59***
	I had a doctor^2^ with a great competence in the field of eating disorders. (14)	.78						.59	.75	PACIC-5A	.42***
	I had a doctor^2^ who cooperated well with my other practitioners. (15)	.61				.31		.50	.77	PACIC-5A	.29*
	After my anorexia was diagnosed, I had regular appointments with a doctor^2^. (12)	.51				.38		.41	.78	PACIC-5A	.42***
	I had a doctor^2^ who arranged that I received appropriate treatment. (8)	.49	.35			.26		.51	.76	PACIC-5A	.24*
	I had a doctor^2^ who recognized my anorexia at an early stage of the illness. (9)	.46	.27	.58				.54	.76	PACIC-5A	.32**
	I had a doctor^2^ who dealt badly with my difficulties concerning food, body-shape or weight (e.g. did not take my complaints seriously or trivialized them).^1^ (11)	.45			− .47			.35	.79	PACIC-5A	.35**
	I had a doctor^2^ who told me unambiguously that I had anorexia. (10)	.43		.35			.33	.48	.77	PACIC-5A	.34**
2 Emotional and practical support from the social environment/ relatives (1.80)	My social environment encouraged me to take up treatment. (6)		.82					.60	.45	F-SozU	.21*
	My social environment often expressed concern about my anorexia. (4)	.25	.72					.43	.64	F-SozU	.43***
	At least one person from my social environment supported me practically in treatment initiation (e.g. arranged or accompanied me to medical appointments). (5)		.71					.46	.64	F-SozU	.14
3 Need and Searching for Orientation and Help (1.60)	It was difficult for me and/ or my relatives or persons I relate to closely to find out whom I could consult best to get help.^1^ (16)			.75	.24			− .16		PACIC-5A	.14
	It helped me to look at or read articles regarding the successful treatment of anorexia or the recovery of other people with anorexia (e.g. in books, reports, social media). (3)	.36		− .57				− .16		BIPQ: Personal control^3^	− .26*
										BIPQ: Treatment control^4^	.23*
										BIPQ: Comprehensibility^5^	.09
4 Social environment recognizing AN and need for help (1.39)	People from my social environment have never addressed my anorexia, or have done so too late.^1^ (1)				.76			.34	.20	F-SozU	.15
	There was at least one person in my social environment who understood at an early stage of the illness that I needed professional help. (2)				.43	.40	.32	.22	.40	F-SozU	.22*
	I believed that undergoing psychotherapy was a sign of weakness.^1^ (17)	.36			.61	.28	.29	.22	.38	BIPQ: Personal control^3^	.14
										BIPQ: Treatment control^4^	.16
										BIPQ: Comprehensibility^5^	.20*
5 Informed relatives (1.22)	My relatives or persons I relate to closely informed themselves on the subject of anorexia (e.g. read books, researched on the internet, visited a counselling center or a doctor^2^). (7)					.82				F-SozU	.05
6 Absence of negative media influence (1.05)	By comparing with girls or women in the media (e.g. television, internet, social media) I considered a certain diet (e.g. a very restricted diet), a very slim figure or a very low weight to be normal so that I didn’t feel the need for treatment.^1^ (18)						.85			BIPQ: Comprehensibility^5^	.20*
										BIPQ: Causal representation “Media” ^6^ ( −)	− .31**
Variance explained (%)		23.33	9.98	8.86	7.71	6.76	5.82				
Cronbach’s alpha		.79	.67	− .38	.42						

^1^The polarity of item 1, 11, 16, 17 and 18 was inverted

^2^Patients are asked in the introduction of the questionnaire, to refer to the physicians they consulted before being referred to specialized care

^3^BIPQ item 3 with 0 = absolutely no personal control and 10 = extreme personal control

^4^BIPQ item 4 with 0 = perception that treatment is not helpful at all and 10 = perception of treatment as extremely helpful

^5^BIPQ item 7 with 0 = no illness perception and 10 = very clear illness perception

^6^BIPQ Item 9, open item asking for the tree major subjective causes for the illness

All illness causes related to influences of media were considered. r^if^ = item-factor correlation, α = Cronbach’s alpha of the component if the item is deleted, (−) negative correlation expected, *p < .05, **p < .01; ***p < .001 (one-sided)

### Construct validity

As hypothesized, patients who reported supportive experiences relating to the health care system perceived more patient-centered, collaborative care prior to treatment initiation, which was reflected by significant correlations with the PACIC-5A total score with 8 out of 9 items of the FABIANA-checklist (with *r* varying from 0.24 to 0.59). The strongest correlations were found for the feeling of trust in the practitioner (item 13, *r* = 0.59, *p* < 0.001), perceived competence of the practitioner and regular contact after AN diagnosis (items 12 and 14, both *r* = 0.42, *p* < 0.001). Moderate correlations were found for the items describing early recognition of AN by the practitioner (item 9, *r* = 0.32, *p* = 0.006) and directly addressing the AN (item 10, *r* = 0.34, *p* = 0.004). Dealing well with patient’s difficulties (not trivializing complaints) (item 11, *r* = 0.35, *p* = 0.003), arranging appropriate treatment (item 8, *r* = 0.24, *p* = 0.029) and cooperating with other practitioners (item 15, *r* = 0.29, *p* = 0.012) were, as expected, related to perceived care. The FABIANA-checklist item 16, indicating difficulties to find out whom to consider for appropriate help, did not correlate significantly with the PACIC-5A score (*r* = 0.14, *p* = *0.134*).

Half of expected relations between items of the FABIANA-checklist that have proximity to the concept of social support and the total score of the F-SozU could be confirmed. The strongest correlation was found for the concerns expressed by the social environment (item 4, *r* = 0.43, *p* < 0.001). Early recognition of the need for treatment by significant others (item 2, *r* = 0.22, *p* = 0.032) and encouragement of the patient to seek help (item 6, *r* = 0.21, *p* = 0.041) were significant at a lower level. The items regarding whether the relatives directly addressed AN (item 1, *r* = 0.15, *p* = 0.100), informed themselves about the AN in the media (item 7, *r* = 0.5, *p* = 0.352) or provided concrete practical support (item 5, *r* = 0.15, *p* = 0.130) were not significantly related to experienced social support.

The expected correlation between consuming media about successful treatments (item 3) and treatment control (BIPQ), i.e. the belief that treatment might be helpful, was confirmed (*r* = 0.23, *p* = 0.028). However, the consumption of media about successful treatments was, contrary to expectations, associated with less personal control (*r* = − 0.26, *p* = 0.015) and showed no significant correlation with illness comprehensibility (*r* = 0.09, *p* = 0.221). Patients who did not perceive undergoing a psychotherapy as a weakness (item 17) had, as expected, a significantly better understanding of their illness (*r* = 0.20, *p* = 0.049, BIPQ), but we found no positive relation to personal control (*r* = 0.14, *p* = 0.124) or treatment control (*r* = 0.16, *p* = 0.084). Patients who reported a lower influence of their weight perception by media (item 18) had, as expected, a better understanding of illness (*r* = 0.20, *p* = 0.042) and named less often media influence as one of the causes of their illness (*r* = − 0.31, *p* = 0.004). An overview on all assumptions on construct validity and corresponding correlations is given in Table 3.

### Average scores of the FABIANA-items

Mean values for all FABIANA-items can be found in Table 2. The items with the highest average scores focused on practical and emotional support from the social environment, e.g. if relatives expressed concern about AN (*M* = 4.2, *SD* = 1.02), perceived the need for help (*M* = 3.6, *SD* = 1.52), informed themselves about AN (*M* = 3.5, SD = 1.38) encouraged the patient to seek treatment (*M* = 4.5, *SD* = 0.98), arranged medical appointments or accompanied the patients to medical consultations (*M* = 4.1, *SD* = 1.41). The average scores indicate, that the primary care provider was more likely not to recognize AN at an early stage (*M* = 2.3, *SD* = 1.40), and that cooperation with other providers was more likely to be less well organized (*M* = 2.5, *SD* = 1.45). Influenced by the media, patients tended to assume that low weight was "normal" or that they did not feel the need for help (*M* = 3.8, *SD* = 1.28). They were more likely to agree that, prior to treatment initiation they felt, that seeking therapy was a sign of weakness (*M* = 3.4, *SD* = 1.43). Differences between adult and adolescent patients were found only for item 7. Relatives of adolescent patients were more likely to inform themselves on the subject of AN or to visit a counselling center (*M* = 4.1, *SD* = 1.11 vs, *M* = 3.3, *SD* = 1.40, *p* = 0.012).

## Discussion

The current study reports on the development and psychometric evaluation of the FABIANA-checklist which aims at assessing factors involved in the process of treatment initiation in patients with AN. The development of the checklist was based on a multi-informant and mixed-method approach. The items of the checklist were derived from facilitators and barriers of treatment initiation that were most frequently named by patients with AN, their relatives and primary care providers in our previous qualitative study [31]. The process of item-selection included item ratings and consensual discussions in the research team, cognitive pre-tests and item-analysis.

Psychometric characteristics of the FABIANA-checklist were tested in a sample of 75 female patients with AN. After item analysis, PCA of the 18-item version of the checklist yielded six components. With Cronbach’s α of 0.77, overall internal consistency was acceptable. The most consistent components regarded the support from the primary health care system and emotional and practical support from relatives or close others. Another yet less consistent component related to the supportive role of relatives in the early recognition of AN and their understanding of the need for treatment. With regard to the other components, the heterogeneity of the items led to unsatisfactory internal consistencies and single item loading. For 14 out of 18 items the expected proximity to well established measures of social support, health care system support, illness perception could be confirmed.

### Perceived support from the primary health care system

The eight items of the first factor are related to the patients’ contact with the health care system before being referred to a specialized treatment. They refer to the practitioners’ competence in dealing with ED, early recognition of the disorder, taking the symptoms seriously and clear communication about the diagnosis. Regular appointments, referral to specialized treatment and the practitioner’s good networking also loaded onto this component. All items of this component showed positive correlations with perceived patient-centered collaborative care, as measured by the PACIC-5A.

Studies show that the majority of patients with AN consult a medical doctor before being referred to specialized care [42, 43]. Often it is in primary care where AN is addressed for the first time and where possible treatment options are discussed [44]. However, previous studies indicate that about 40% of practitioners do not correctly identify AN and only 26–40% of patients with AN-diagnosis are referred to specialized treatments by their practitioners [11, 45]. Patients with AN are particularly adept at hiding their weight and body shape from their medical doctor. Nevertheless, empirical evidence suggests that the majority of practitioners do not regularly screen for ED or believe to lack the necessary skills to intervene properly in ED [46] due to fear of patients’ defensiveness or insecurity about which questions to ask. The authors hypothesized that medical doctors may be adopting a "watchful waiting" approach, which is frequent when it comes to diagnosing mental illness in primary care [47]. However, even if patients with AN are referred to specialized treatment, this does not guarantee that they will start or complete a psychotherapeutic treatment [48, 49]. Hay et al. [50] showed that the subsequent use of specialized psychiatric treatment was influenced by whether or not mental health issues were raised in primary care visits.

Previously developed checklists and reviews on treatment barriers usually include some factors related to the health system [6, 25, 28, 29, 51]. However, these factors mainly address general aspects, such as difficulties in accessing specialized care, long waiting times or high treatment costs. The FABIANA-checklist focusses more on the practitioner’s specific approach to the disorder in the frame of a doctor-patient relationship and networking with colleagues. From the responses of the patients in our sample, there are indications that the early treatment of AN by the practitioner and pathways to specialized care may be further optimized. While patients quite often stated that they had a doctor they could trust, they less often agreed with the items related to their doctor's competence in dealing with ED, organizing cooperation with other treatment providers or dealing directly with their AN. The recognition and competent management of AN within the health care system therefore seems to be an important starting point for preventive measures. It is important to note that the FABIANA-checklist does not refer exclusively to contacts with general practitioners but to any contact with the medical care system that took place prior to referral for specialized treatment.

### Support from the relatives and close others

The second factor refers to three items which describe the perceived support from relatives and close others. Correlations between the items and the F-SozU [37], a validated measure for social support, were all positive. The correlation with item 5 was however too small to be significant. While the F-SozU captures more general aspects of social support, item 5 refers to a quite specific assistance in initiating treatment, which was even further specified by presenting examples e.g. e close relatives “arranged doctor's appointments” or “accompanied me to medical appointments”. It is possible that the correlation would have been larger without this further restriction through examples.

Social support is mentioned in the checklist on treatment barriers proposed by Ali et al. [29] and in existing systematic reviews on barriers to help-seeking behavior [51, 52]. While the BATSH-ED refers to a general lack of support or encouragement from others or to the inability of others to provide help, the FABIANA-checklist differentiates the relatives' concern for the patient, the encouragement of the patient to seek treatment and concrete practical support. Barriers and helpful factors are not necessarily comparable in their influence, even if they are endpoints on the same spectrum. For example, Cachelin et al. [52] reported that in 23–35% of patients with ED, encouragement from friends was a major facilitator for entering treatment. However, a lack of support was perceived by only 3% as a barrier.

In our sample patients agreed particularly often with factors of emotional and practical support. Moreover, the item on early detection of AN by relatives was on average more likely to be endorsed than the item referring to early detection of AN by practitioners. Although no causal conclusions can be drawn from our data, this indicates that close others play an important role in early detection and in supporting the patient on her way to specialized care.

Three items mainly loaded onto the fourth component, which, however, had insufficient internal consistency. One item refers to an inner attitude of the patient (perceiving the need of psychotherapy as a sign of weakness) and two items address the early recognition of AN and the need for help by close others. This component might be related to some extent to aspects of stigmatization and shame, which have been found to be potential barriers to treatment initiation in other studies [25, 28, 51]. On the one hand, it could be assumed that patients who consider therapeutic help as a sign of personal weakness, might feel more embarrassed and ashamed when personal problems occur and might be more afraid of being blamed by others. Thus, they might hide their problems from their relatives or close others, which in turn will present more difficulties in recognizing and addressing their need for help. On the other hand, dealing openly with weaknesses and disorders in the family (or in society) can make it easier for the patient to confide in others and to get the necessary support from close relatives. Interestingly, there was no general correlation between relatives addressing AN (item 1) and the total score of perceived social support. But the item was correlated with a single FSozU item, namely with the feeling of (the patient) of being able to share happiness and sorrow with close others.

### Further components

The other components were characterized by either negative internal consistency or single item loadings. These components describe similar topics, namely knowledge about AN and available treatments and the consideration of role models in the period prior to treatment uptake. Ali et al. [29] describe a lack of knowledge about the ED as a barrier to help-seeking behavior and mention a factor about knowledge about where to get help and about which treatments are available, which coincides with item 16 of the FABIANA-checklist. This item could not be validated with the PACIC-5A [36] as expected. It probably refers more to a general feeling of orientation, in the sense of knowing where to search for help, rather than to have perceived concrete support from the health system.

In addition to the patients' level of knowledge, the FABIANA-checklist also recorded whether any relatives had informed themselves about AN. In this item (7) differences between adolescent and adult patients emerged, with the relatives of adolescents searching more frequently for advice or information about AN. In contrast to our expectations this item was not correlated to general social support. It probably refers more to the support that relatives seek for themselves in literature or counselling in order to be able to in turn support patients.

Item 3 was retained in the checklist despite having an item-total correlation close to zero, because it represented an aspect that was not represented by any other item, namely the orientation towards other patients with successful therapy courses (as role models). This item, which did not correlate significantly with any other item of the checklist, complicated the interpretation of the PCA. Contrary to our expectation, it had a negative impact, contributed to a negative Cronbach's alpha on the third component and was neither associated with more personal control nor with more illness comprehensibility. We were not able to fully interpret the third component. This should be seen as an indication that this item needs be examined more closely and, if necessary, excluded from the checklist.

Patients with AN confirmed that comparing themselves to other girls or women in the (social) media had an impact on the fact that they considered their low weight or their dieting as normal and that they subsequently did not felt the need to seek treatment. Patients who were less influenced by the media had, as expected, a better understanding of their disease and were also less likely to perceive the media as a cause of their illness in the BIPQ [38].

## Strengths and limitations

To the best of our knowledge, the FABIANA-checklist is the only checklist of factors involved in treatment initiation in patients with AN that underwent psychometric validation.

In contrast to a classic questionnaire, which is based on theoretical concepts, the FABIANA-checklist was created using a mixed-method and a multi-informant (patients, their relatives and practitioners) approach, and is thus summarizing aspects that were considered relevant to the agents involved in treatment initiation. The use of a bottom-up approach (based on qualitative interviews) ensures the external validity of our checklist. Through the inclusion of the most frequently mentioned categories it is ensured that the factors listed in the checklist address a broad group of patients with AN. Even though it was important to us to collect a broad range of factors, we were unable to include all aspects mentioned in the interviews. For reasons of parsimony and applicability of the checklist, we had to exclude factors that were mentioned by only a few patients or that were very similar to other factors, even if they covered slightly different aspects. Moreover, even if we had a large qualitative sample, it is possible that aspects were not covered or were underrepresented in our sample.

Another strength is the process of item selection, which was guided by consensual rating discussions within the research group and a focus on items that are potentially modifiable by secondary prevention measures.

The sample of our study is representative of female patients with AN. The decision to include only female patients with AN was based on the fact, that women are by far the most frequently affected patient group. In addition, it is possible that other factors are involved in men's treatment uptake [53]. However, in order to study gender specific barriers and facilitators, we would have had to collect a much larger sample, which would have demanded a lot of time and resources. In addition, since the preliminary study was based exclusively on interviews with female patients, we assume that the factors contained in the checklist can only be transferred to a limited extent to a male target group.

As the survey was conducted at 11 sites, the sample is fairly representative of patients with AN undergoing specialized treatment in Germany. Since the large majority of patients was recruited at inpatient units, these patients are overrepresented in the sample. With an average BMI of 15.5 kg/m^2^ and a comorbidity rate of 77%, the analyzed population is comparable to other studies [6, 54]. We found a mean DUI of 2.25 years for female patients with AN. The DUI is slightly below the mean DUI for patients with AN from seven countries described by Austin et al. [6] in their recent systematic review (2.42 years), and similar to another German sample from the Hamburg metropolitan area [44]. In the subgroup of adolescent patients, DUI was a little less than 6 months and thus significantly shorter than in adults. Mean AOO was around 19 years, with 15.5 years for adolescent patients and 20.7 years for adults. The shorter DUI found in adolescent patients has been demonstrated in other studies [6, 42, 47] and can be attributed to significantly lower dispersion in the subsample of adolescents. Moreover, other studies suggest, that adolescents are more likely living with their relatives, who support the recognition and help-seeking process.

With regard to the sample size, it can be said that approximately 3 participants per item is acceptable but in the lower range of the recommendations for PCAs. It is therefore possible that the PCA was underpowered.

Finally, the FABIANA-checklist explicitly refers to the time that preceded the first psychotherapeutic treatment, it will not allow conclusions on correlates of treatment-seeking in general. Moreover, retrospective memory creates distortions, i.e. some factors are experienced as more significant or less significant than they were at the given time. The literature provides no sufficient evidence on the optimal recall period to minimize the impact of memory effects (e.g., recall biases) for self-reported utilization of healthcare services [55]. However, it is recommended to use periods of three or six months when frequently used services are surveyed while salient visits and rarely used medical care services seem to be accurately reported over a longer period [55]. We assume the commencement of a psychotherapeutic treatment to be a salient and rare event, justifying the use of a 12-month-period for our study purposes.

## Conclusion

The FABIANA-checklist is a psychometrically evaluated instrument, which specifically refers to factors involved in treatment initiation in patients with AN. The 18-item version has an acceptable internal consistency and due to the bottom-up approach a high external validity. The present study provided initial data on the expression of these factors in a population of patients who were currently or recently undergoing their first specialized AN treatment. As the focus of the FABIANA-checklist has been laid on modifiable factors, it offers a good starting point for the overall aim of the FABIANA-project, i.e. deriving preventive measures. To quantify the relative importance of the factors assessed by the FABIANA-checklist and to derive recommendations on early-intervention approaches, the effect of these factors on the DUI will be determined in our upcoming study. Furthermore, we plan to investigate differences between the perspectives of patients, their close relatives and primary caregivers on factors involved in the process of treatment initiation.

## Supplementary Information

**Additional file 1.** Development of the FABIANA-checklist including item construction, item rating and item selection.

## Data Availability

Psychometric data can be made available by the authors upon legitimate request.

## Abbreviations

AN: Anorexia nervosa
AOO: Age of onset
BATSH-ED: Barriers toward seeking help for eating disorders questionnaire
BED: Binge eating disorder
BIPQ: Brief illness perception questionnaire
BN: Bulimia nervosa
DUI: Duration of untreated illness
ED: Eating disorder
FABIANA: Facilitators and barriers in anorexia nervosa treatment initiation
FREED: First episode and rapid early intervention in eating disorders
F-SozU: Social Support Questionnaire (Fragebogen der sozialen Unterstüztung)
PACIC-5A: Patient assessment of chronic illness care questionnaire
