# Supplementary material for: The development and psychometric evaluation of FABIANA-checklist: a scale to assess factors influencing treatment initiation in anorexia nervosa
Source: J Eat Disord. 2021 Nov 3;9:144. doi: 10.1186/s40337-021-00490-w (PMC8565008; doi:10.1186/s40337-021-00490-w)
Supplement: Supplementary file 1 — Additional file 1. Development of the FABIANA-checklist including item construction, item rating and item selection. [file 40337_2021_490_MOESM1_ESM.docx]

**Additional file 1: Development of the FABIANA-checklist including item construction, item rating and item selection.**

|  |  | Item rating | | | |  | Item selection | | |
| --- | --- | --- | --- | --- | --- | --- | --- | --- | --- |
| Facilitators and barriers [31] | Item construction (N=73) | Research team  rating (N=4) | Cognitive pre-tests  Patients (N=9) | | |  | a | b | Final items |
|  |  | *M* | Rel *M* | Com *M* | Rec M |  |  |  |  |
| (Not) recognizing and addressing | People from my social environment have never addressed my anorexia, or have done so too late. | 3.00 | 2.33 | 2.78 | 2.56 |  |  |  | Item 1 |
|  | The people around me did not recognize my anorexia, or have done so too late. | 2.50 |  |  |  |  |  |  |  |
|  | People around me have opened my eyes. | 1.00 |  |  |  |  |  |  |  |
| (No/wrong) recommendation and referral | My doctor(s) ensured that I got the right treatment. | 1.25 |  |  |  |  |  |  |  |
|  | I had a doctor who arranged that I received appropriate treatment. | 2.75 | 2.33 | 2.78 | 3.00 |  |  |  | Item 8 |
|  | I was treated only by doctors who do not offer psychotherapy for too long (e.g. general practitioner, pediatrician, psychiatrist). | 2.75 |  |  |  |  |  |  |  |
|  | For too long, solely my physical symptoms were treated. | 2.25 |  |  |  |  |  |  |  |
|  | After my anorexia was diagnosed, I was in medical treatment for a long time without getting a clear recommendation for psychotherapy. | 2.25 | 1.56 | 2.67 | 2.89 |  |  |  |  |
|  | I have often received vague or noncommittal recommendations only from doctors. | 2.75 | 1.44 | 2.78 | 3.00 |  |  |  |  |
|  | My doctors have often given me vague or noncommittal recommendations only. | 2.00 |  |  |  |  |  |  |  |
| Competence, specialization, training | I had a doctor who is competent in dealing with female patients with eating disorders. | 2.50 |  |  |  |  |  |  |  |
|  | I had a doctor with high competence in the field of eating disorders. | 2.75 | 2.11 | 3.00 | 3.00 |  |  |  | Item 14 |
|  | My doctor is a specialist in eating disorders. | 2.00 |  |  |  |  |  |  |  |
|  | I had a doctor who is competent to address both physical and psychological problems. | 1.75 |  |  |  |  |  |  |  |
|  | I would have preferred to go to a specialized hospital for consultation. | 1.25 |  |  |  |  |  |  |  |
| (Non) understanding of AN as illness or the need for treatment | My social environment did not understand my illness (e.g. "Just start eating again"). | 3.00 | 1.56 | 2.89 | 2.78 |  |  |  |  |
|  | Those around me did not understand the need for treatment for too long. | 2.50 |  |  |  |  |  |  |  |
|  | There was at least one person in my social environment who understood at an early stage of the illness that I needed professional help (e.g. from a doctor, a psychotherapist or an counselling center). | 3.00 | 2.11 | 2.78 | 2.78 |  |  |  | Item 2 |
|  | People around me who also have difficulties with food, weight, or body-shape made it harder for me to start treatment. | 1.25 |  |  |  |  |  |  |  |
| Health education and de-stigmatization of AN and psychotherapy | I had the feeling that others were judging people undergoing psychotherapy. | 1.00 |  |  |  |  |  |  |  |
|  | I worried about being judged by others if I would undergo psychotherapy. | 1.25 |  |  |  |  |  |  |  |
|  | I believed that undergoing psychotherapy was a sign of weakness. | 2.75 | 2.33 | 3.00 | 3.00 |  |  |  | Item 17 |
| Exchange, support, concern, understanding | My social environment often expressed concern about my anorexia. | 3.00 | 2.44 | 2.89 | 3.00 |  |  |  | Item 4 |
|  | The people around me kept a clear head but pointed out that I needed professional help. | 2.50 |  |  |  |  |  |  |  |
|  | It helped me to exchange ideas with people who had already undergone psychotherapy. | 3.00 | 1.89 | 2.89 | 2.89 |  |  |  |  |
| Positive role models for treatment | I have looked at reports or read articles about the treatment of other patients with anorexia (e.g. in books, reports, social media). | 2.25 |  |  |  |  |  |  |  |
|  | It helped me to look at or read articles regarding the successful treatment of anorexia or the recovery of other people with anorexia (e.g. books, reports, social media). | 3.00 | 1.56 | 2.89 | 3.00 |  |  |  | Item 3 |
|  | People around me reported that they themselves are or have been in psychotherapy. | 1.50 |  |  |  |  |  |  |  |
|  | It was helpful to hear that people around me themselves are or have been in psychotherapy. | 2.25 |  |  |  |  |  |  |  |
|  | I have exchanged information with others who have been in psychotherapy. | 2.50 |  |  |  |  |  |  |  |
|  | It was encouraging to me to learn that psychotherapy has helped other people. | 1.50 |  |  |  |  |  |  |  |
| (No) reminding of, making of or accompanying to appointments | At least one person from my social environment supported me practically in treatment initiation (e.g. arranged or accompanied me to medical appointments). | 2.75 | 2.44 | 2.67 | 3.00 |  |  |  | Item 5 |
|  | My social environment has motivated me to keep the appointments I made. | 1.50 |  |  |  |  |  |  |  |
| Suggesting or encouraging to seek treatment | My social environment encouraged me to take up treatment. | 2.00 | 2.56 | 3.00 | 3.00 |  |  |  | Item 6 |
|  | My social environment has told me that it is right for me to start treatment. | 1.75 |  |  |  |  |  |  |  |
| Somatic symptoms / Exacerbation and personal breaking point reached | I was only ready for treatment as my (physical or mental) breaking point was reached. | 2.50 | 2.67 | 2.89 | 3.00 |  |  |  |  |
|  | Without my physical symptoms and complaints, I would not have undergone psychotherapy. | 2.50 | 2.67 | 3.00 | 2.78 |  |  |  |  |
|  | I only took up treatment because of my physical symptoms and complaints. | 2.25 |  |  |  |  |  |  |  |
| Networking, cooperation | I had a doctor who collaborated well with my other practitioners (e.g. other doctors, psychotherapists). | 2.75 | 1.44 | 2.78 | 3.00 |  |  |  | Item 15 |
| (No/vaguely) diagnosing or communicating diagnosis | My doctors never recognized anorexia or didn't recognize it for too long. | 2.00 |  |  |  |  |  |  |  |
|  | I had a doctor who recognized my anorexia at an early stage of the illness | 3.00 | 1.44 | 2.89 | 3.00 |  |  |  | Item 9 |
|  | My doctors never informed me that my symptoms belonged to anorexia, or did so too late. | 2.00 |  |  |  |  |  |  |  |
|  | I had a doctor who informed me that my symptoms were typical for anorexia nervosa. | 2.75 | 1.22 | 3.00 | 3.00 |  |  |  |  |
|  | I had a doctor who told me unambiguously that I had anorexia. | 3.00 | 1.67 | 3.00 | 3.00 |  |  |  | Item 10 |
|  | Doctors or therapists have missed my difficulties with food, body-shape or weight. | 1.50 |  |  |  |  |  |  |  |
|  | My difficulties with eating, body-shape or weight have often been missed by doctors or therapists. | 2.00 |  |  |  |  |  |  |  |
| Trivializing and neglected assistance | I often had bad experiences addressing my difficulties regarding eating, body-shape or weight with doctors or therapists (e.g. not taking complaints seriously or playing them down). | 2.00 |  |  |  |  |  |  |  |
|  | I had a doctor who dealt badly with my difficulties concerning food, body-shape or weight (e.g. did not take my complaints seriously or trivialized them). | 3.00 | 1.89 | 3.00 | 2.89 |  |  |  | Item 11 |
|  | I have seen a doctor or therapist who did not take my difficulties concerning food, body-shape or weight seriously. | 2.75 |  |  |  |  |  |  |  |
|  | I have often experienced that doctors or therapists do not take my difficulties with eating, body-shape or weight seriously. | 2.00 |  |  |  |  |  |  |  |
|  | I often experienced that doctors and therapists handled my difficulties regarding eating, body-shape or weight badly (e.g. not taking complaints seriously or playing them down). | 2.00 |  |  |  |  |  |  |  |
|  | As a result of my bad experiences with doctors or therapists I initially wanted to manage the problem alone. | 1.50 |  |  |  |  |  |  |  |
| (Reducing) comparisons with media ideals | I did not understand why I should seek treatment for something that is presented as normal in the media. | 2.00 |  |  |  |  |  |  |  |
|  | By comparing with girls or women in the media (e.g. television, internet, social media) I considered a certain diet (e.g. a very restricted diet), a very slim figure, or a very low weight to be normal so that I didn’t feel the need for treatment. | 2.25 | 2.78 | 2.00 | 3.00 |  |  |  | Item 18 |
| Continuity (of care) and regular control examinations | After my anorexia was diagnosed, I had regular appointments with a doctor. | 3.00 | 1.56 | 3.00 | 3.00 |  |  |  | Item 12 |
|  | My anorexia was diagnosed during a routine medical examination. | 1.25 |  |  |  |  |  |  |  |
| Concerns regarding treatment-caused absence in school, work or childcare | I was afraid that other things in life (e.g. school, job, children) would have to stay behind because of the treatment. | 2.00 |  |  |  |  |  |  |  |
|  | I was afraid that the treatment would not be compatible with other things important to me (e.g. school, job, children). | 3.00 | 2.22 | 2.89 | 3.00 |  |  |  |  |
| Information policy and the professional web presence | Among the large number of offers, it was difficult to find a suitable treatment. | 3.00 | 2.22 | 3.00 | 2.78 |  |  |  |  |
|  | It was difficult for me and/or my relatives or persons I relate to closely to find out whom I could consult best to get help. | 3.00 | 2.11 | 3.00 | 3.00 |  |  |  | Item 16 |

| (No) positive patient-physician relationship | I had trust in and a good relationship with my doctors. | 1.50 |  |  |  |  |  |  |  |
| --- | --- | --- | --- | --- | --- | --- | --- | --- | --- |
|  | I had a doctor I trusted. | 3.00 | 1.56 | 2.89 | 3.00 |  |  |  | Item 13 |
|  | I had a good relationship with my doctor(s). | 1.50 |  |  |  |  |  |  |  |
|  | I had a doctor with whom I had a good relationship. | 3.00 | 1.89 | 3.00 | 3.00 |  |  |  |  |
|  | I felt supported by my doctors. | 1.75 |  |  |  |  |  |  |  |
|  | I had a doctor through whom I felt supported with my difficulties with food, body-shape or weight. | 1.50 |  |  |  |  |  |  |  |
| Delays due to necessary referral letters, medical reports or cost agreements | I had to do many preliminary examinations with doctors before I could start my treatment. | 1.50 |  |  |  |  |  |  |  |
|  | The beginning of my treatment was delayed because I had to obtain many preliminary findings (e.g. results of physical examinations, preliminary medical reports). | 1.75 | 1.00 | 2.78 | 3.00 |  |  |  |  |
| Relatives inform themselves about AN and treatment options | My relatives or persons I relate to closely informed themselves on the subject of anorexia (e.g. read books, researched on the internet, visited a counseling center or a doctor). | 1.75 | 1.56 | 3.00 | 3.00 |  |  |  | Item 7 |
|  | Supported by relatives or persons I relate to closely, I was able to consider which treatment might be the right one. | 1.25 |  |  |  |  |  |  |  |
| Pressure and reproaches | My environment has blamed me for my anorexia. | 2.00 |  |  |  |  |  |  |  |
|  | At least one person from my social environment has frequently blamed me for my anorexia nervosa. | 2.25 | 1.78 | 3.00 | 2.89 |  |  |  |  |
|  | My environment has put pressure on me. | 1.25 |  |  |  |  |  |  |  |

The rating provided by the research included comprehensibility, clarity and modifiability of the items (composite score). The patients’ rating (cognitive pre-tests) included relevance (Rel), comprehensibility (Com) and recallability (Rec). Items were rated on a 3-point scale from 1 = low to 3 = high. *M*=Mean score. Items selected for cognitive pre-testing (N=30) are shaded in grey. The length of the bars refers to the selection process with a= exclusion after cognitive pre-tests (N=5) and b=exclusion after item analysis (N=7), leading to the final version of the FABIANA-checklist with 18 items
